# Crosstalk between bile acids and gut microbiota: a potential target for precancerous lesions of gastric cancer

**DOI:** 10.3389/fphar.2025.1533141

**Published:** 2025-03-13

**Authors:** Maofu Zhang, Jialin Zhong, Yanyun Shen, Zhongyang Song

**Affiliations:** ^1^ Clinical College of Traditional Chinese Medicine, Gansu University of Chinese Medicine, Lanzhou, Gansu, China; ^2^ Department of Oncology, Affiliated Hospital of Gansu University of Chinese Medicine, Lanzhou, Gansu, China

**Keywords:** precancerous lesions of gastric cancer (PLGC), bile acids (BAs), gut microbiota, crosstalk mechanism, traditional Chinese medicine

## Abstract

As a critical juncture in the pathological continuum from gastritis to gastric cancer, precancerous lesions of gastric cancer (PLGC) are increasingly prevalent, significantly undermining the health of the global population. The primary constituents of bile, specifically bile acids (BAs), disrupt the equilibrium of gastric hormone secretion and compromise the structural integrity of the gastric mucosa, thereby facilitating gastric oncogenesis. Moreover, gut microbiota modulate host physiological and pathological processes through immune response regulation, metabolic pathway interference, and direct interaction with gastric tumor cells. Extensive research has elucidated that the metabolic dysregulation of BAs and gut microbiota, in concert with the resultant impairment of the gastric mucosa, are central to the pathogenesis of PLGC. In anticipation of future clinical preventive and therapeutic strategies, this review collates recent insights into the roles of BAs and gut bacteria in PLGC, examining their interplay and significance in the pathogenic mechanism of PLGC.

## Graphical Abstract

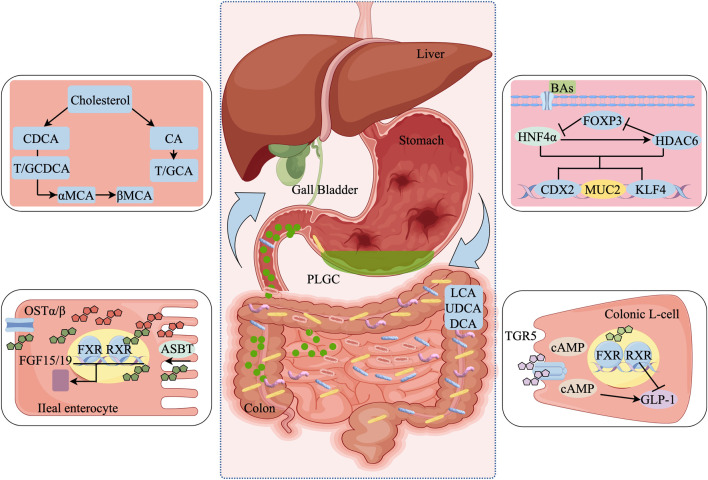

## 1 Introduction

Globocan-2020 based on the data of the World Health Organization shows that gastric cancer occupies the fifth place in the frequency of cancer diseases, accounting for 5.6% of all new cases of malignant neoplasms (Zhang et al., 2025). This was observed especially in China where the new cases of GC stood at 478,508 in a single year, which was 43.9% of the global statistics (Ye et al., 2025). GC related deaths make up 7.7% of all cancer deaths and GC is the fourth most lethal cancer worldwide. Of these deaths, 373,789 are reported to happen in China, which makes up 48.6% of the total global deaths (Conti et al., 2023; Yang T. et al., 2023). Such statistics indicate a dire importance of paying more attention to early diagnosis and better treatment regimens in the clinical oncology research. The PLGC are particularly important because they are the stage that is considered as the “golden turning point” in the development from gastritis to gastric cancer. These changes which are associated with an increased risk of malignancy are usually presented through a range of dyspeptic complaints including gastric pain, bloating and epigastric discomfort. The assessment of such conditions mainly involves the use of gastroscopy and histopathological biopsies (Moon et al., 2024; Sun et al., 2024; Zhang M. et al., 2024). According to the internationally acclaimed Correa model, the development of GC progresses through several stages: starting from chronic superficial gastritis, through chronic atrophic gastritis (CAG), and intestinal metaplasia (IM) and ending with dysplasia (Dys). Importantly, the last three stages are subclasses of PLGC (Guo et al., 2024; Wang B. et al., 2024; Xi et al., 2023). A screening study shows that CAG was found to be present in 25.8%, IM in 23.6%, while Dys was found in 7.3% of the sample and all of these have been seen to be on the rise (Du et al., 2014). Therefore the careful evaluation and management of these precancerous conditions and lesions are now amongst the most active areas of research into strategies for the early prevention and control of this type of gastric cancer (Cao et al., 2023).

*Helicobacter pylori (H. pylori*) has been confirmed as one of the main pathogens which are responsible for the development of PLGC as highlighted in (Mülder et al., 2024; Peng et al., 2023; Zhu et al., 2023). This pathogen targets the gastric epithelial cells and causes cell damage together with the release of various enzymes and factors that facilitate pathogenicity. These substances start complex immune and inflammatory processes which are involved in the pathogenesis of the process. Also, *H. pylori* has been associated with reduction in the density of gastric glands in the lamina propria, alteration of the structure of the stomach, and may also contribute to both intestinal epithelial hyperplasia and atypical hyperplasia. These conditions if not well managed can progress to full blown gastric cancer (Zeng et al., 2024). In addition, bile reflux into the stomach, which is the main factor of exposure of the gastric mucosa to bile, contains Bile acids (BAs). These acids are able to directly injured the lipoproteins of the mucosa thus exposing the gastric lining. At the same time, BAs enhances the secretion of gastric acid from the gastric mural cells worsening the injury of gastric mucosa and thus promoting the occurrence of PLGC (Li et al., 2020; Wang et al., 2023; Yang et al., 2024). Other factors that have been reported to affect the development of PLGC include the inflammatory mediators, genetic factors, psychosocial stressors and diet patterns which are known to have a close relationship with the advancement of this condition (Tan et al., 2020).

At present, the diagnostic procedure of PLGC includes the application of gastroscopy, biopsy and the examination of certain blood indices. The main strategy of intervention is to eliminate *H. pylori* infection through methods like endoscopic mucosal resection and through the use of proton pump inhibitors among others (Duan et al., 2024; Xu et al., 2022; Zhang et al., 2023). PLGC is caused by the following: inflammation, autophagy, apoptosis, glyclysis and ferroptosis. While recent advances in therapy have provided new ideas and techniques for the treatment of patients, the emphasis in research has been to examine these processes in isolation, (Yang W. J. et al., 2023; Zhang et al., 2018; Zhang N. et al., 2024). The study has shown that alteration of the metabolic balance between BAs and the gut microbiota affects the inflammatory tone in the gastric mucosa and thus the advancement of PLGC. BAs are derived from cholesterol and are produced in the liver, they are further metabolized in the small intestine and a small proportion in the colon throughout an interaction with the gut microbiome. This biotransformation is mediated by several receptor pathways such as farnesoid X receptor (FXR) and Takeda G protein-coupled receptor 5 (TGR5) that are very important in determining the signaling properties of BAs (Wahlström et al., 2016). In addition, BAs are capable of modulating the inflammatory signaling pathways either by acting on the gut microbiota or by inducing the activation of innate immune genes in the small intestine. Previous work has also confirmed that the mutual interactions between BAs and gut microbiota are involved in the formation of inflammation and tumorigenesis in the gastrointestinal tract. Furthermore, the application of herbal ingredients and components appears to have potential for improving PLGC through inclined regulation of the balance between BAs synthesis and gut microbiota to regulate the gastric environment. Therefore, this paper presents a comprehensive synthesis of the most recent studies on the role of BAs and gut microbiota in PLGC with the focus on the mechanisms of their interaction and the description of their exact functions in the disease progression.

## 2 Progress of BAs research in the context of PLGC

### 2.1 Overview of BAs regulatory mechanisms

BAs are synthesized as amphiphilic, water-soluble molecules, deriving from cholesterol within hepatocytes. These molecules include saturated hydroxylated C-24 sterols, which are produced exclusively by the liver, the only organ equipped with all 14 enzymes required for BAs synthesis (Liu et al., 2024). The primary BAs synthesized in both humans and rodents are cholic acid (CA) and chenodeoxycholic acid. In rodent species, a substantial conversion of CDCA into methylcholic acid occurs through 6β-hydroxylation, a metabolic transformation that is absent in human biochemistry (Zhang et al., 2014). Following synthesis, these primary BAs are converted through N-acyl amidation into glycine or taurine conjugates, leading to the formation of conjugated BAs such as taurocholic acid in humans. This conjugation process significantly enhances the solubility of BAs, particularly under physiological pH conditions where only a scant fraction of non-conjugated BAs can dissolve. The ratio of glycine to taurine conjugated BAs varies significantly across different mammalian species. These conjugated forms are then actively secreted across ductal cell membranes into the bile ducts, subsequently flowing into the gallbladder. Besides BAs, bile itself comprises a complex mixture of substances including phospholipids, bilirubin, immunoglobulin A, mucus, and a variety of endogenous products. In the gallbladder, bile undergoes a 5–10 fold concentration *via* the removal of water and electrolytes and is acidified by a Na^+^/H^+^ exchanger. In general, approximately half of the bile produced is stored in the gallbladder, with the remainder bypassing it to flow directly into the gastrointestinal tract. The release of concentrated bile into the proximal gastrointestinal tract is triggered by cholecystokinin, a hormone secreted from the duodenum during meals, which stimulates the gallbladder to contract (Winston and Theriot, 2020).

**FIGURE 1:**
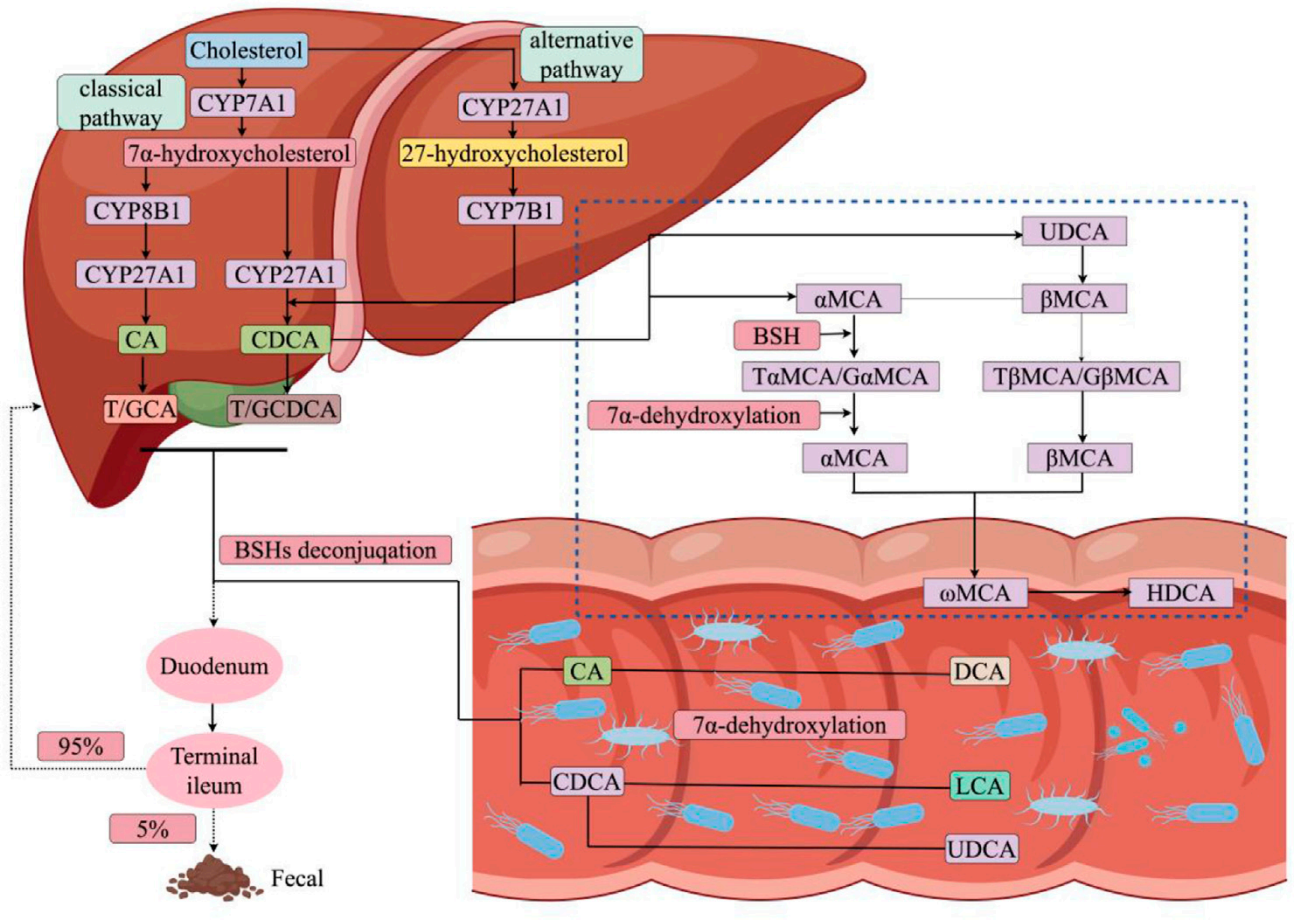
BAs regulatory mechanisms. Primary BAs is produced from cholesterol *via* the CYP7A1-mediated classical or CYP27A1-mediated alternative pathway. Subsequently, BAs and Chenodeoxycholic acid (CDCA) combine with glycine or taurine to form conjugated BAs, which are excreted through the bile ducts into the intestine. Once released into the gut, these BAs are altered by the gut microbiome, leading to secondary BAs production, and approximately 95.0% of the BAs is reabsorbed in the terminal ileum, allowing it to be circulated by the liver. CYP7A1, cholesterol 7α-hydroxylase; CYP27A1, recombinant cytochrome P450 27A1; CYP8B1, recombinant human 7-alpha-hydroxycholest-4-en-3-one 12-alpha-hydroxylase; CYP7B1, porcine cytochrome P450 7B1; CA, cholic acid; T/GCA, taurocholic acid/glycocholic acid; T/GCDCA, taurochenodeoxycholic acid/glycochenodeoxycholic acid; BSHs, Bile Salt Hydrolase; UDCA, ursodeoxycholic acid; αMCA, α-Muricholic acid; βMCA, β-Muricholic Acid; ωMCA, ω-Muricholic Acid; HDCA, hyodesoxycholic acid; DCA, deoxycholic acid; LCA, lithocholic acid.

In the liver, BAs synthesis occurs *via* two distinct pathways: the classical and the alternative (Xing et al., 2023; Yang et al., 2023) (Figure 1). Under normal conditions, the classical pathway accounts for at least 75% of BA production and is mainly initiated and determined by cholesterol 7α-hydroxylase (CYP7A1). This pathway commences with the modification of the cholesterol molecule by introducing a hydroxyl group at the C-7 position to produce 7α-hydroxycholesterol (Chiang and Ferrell, 2022). Subsequently, this compound is subject to a double-bond isomerisation and 3β-hydroxy oxidation. These reactions are catalyzed by NAD-dependent microsomal enzymes, specifically the 3β-hydroxyl oxidation-Δ5-C27-steroid dehydrogenase/isomerase, resulting in the formation of 7-α-hydroxy-4-cholesten-3-one (C4). The transformation of C4 into other BAs is a rate-limiting step that involves various enzymes, including sterol 12α-hydroxylase (CYP8B1), which directs the synthesis towards CA. Conversely, in the absence of CYP8B1, enzymes such as aldo-keto reductases AKR1D1 and AKR1C4 act on C4, leading to the synthesis of CDCA. The alternative pathway, accounting for about 9.0% of BAs synthesis in human hepatocytes, is initiated by mitochondrial sterol 27-hydroxylase (CYP27A1). This enzyme catalyzes the oxidation of cholesterol into 27-hydroxycholesterol and 3β-hydroxy-5-cholestenoic acid. The latter metabolites, following hydroxylation by oxysterol 7α-hydrolase, become 7α,27-dihydroxycholesterol and 3β,7α-dihydroxy-5-cholestenoic acid, serving as precursors in the synthesis of CDCA, the predominant BAs in the alternative pathway, unlike the classical pathway which generates both CDCA and CA (Chiang and Ferrell, 2019).

### 2.2 BAs regulates the PLGC mechanism

BAs are recognized not only as biomarkers for the early detection of GC but also as independent risk factors for PLGC. Research indicates that BAs contribute to PLGC pathogenesis through various mechanisms such as compromising the integrity of the gastric mucosa, altering the equilibrium of gastric hormone secretion, and influencing the gastric microbial landscape (Fu et al., 2022; Zhang et al., 2021). In a comprehensive multicentre clinical study, a strong correlation was established between elevated BAs reflux levels into the stomach and an increased risk of CAG and IM (Matsuhisa et al., 2013). Additionally, in gastric epithelial cells, BAs exposure has been shown to trigger the upregulation of miR-92a-1-5p. This upregulation leads to the suppression of Forkhead Box Protein D1 (FOXD1) and the prolonged activation of the NF-κB signaling pathway, which subsequently enhances the transcription of the caudal type homeobox transcription factor 2 (CDX2) and promotes intestinal differentiation. Thus, strategies focusing on the inhibition of miR-92a-1-5p and the restoration of FOXD1 expression may offer viable therapeutic interventions (Li et al., 2019).

Repeated incidents of bile reflux are strongly linked to a higher risk of developing IM, which emphasizes the significant role of bile reflux in the pathogenesis of PLGC (Lei et al., 2023). Experimental models utilizing DCA to induce CAG have revealed that DCA contributes to the disruption of the gut microbiota, damages the mucosal barrier, and leads to immune system dysregulation. Importantly, DCA increases the population of enterococci and cyanococci within the gastrointestinal tract. This change in microbial composition further facilitates the transformation of BAs, amplifying the cytotoxic effects of DCA and lithocholic acid (LCA), which are among the more potent fat-soluble BAs (Cheng et al., 2024). Additionally, investigations involving patients positive for *H. pylori* have shown a robust positive correlation between the levels of BAs and the presence of CAG and IM. BAs treatments have been observed to significantly upregulate the expression of CDX2 in RGM-1 gastric epithelial cells. In these cellular environments, co-culture with *H. pylori* markedly boosts the expression of the cytokine-induced neutrophil chemoattractant 1 (CINC1). However, the application of glycyl DCA has been shown to mitigate this effect, highlighting the potential of BAs to promote the development of PLGC in environments devoid of inflammatory cell infiltration (Tatsugami et al., 2012).

In their research, Xu et al. administered varying concentrations of BAs to normal gastric epithelial cells (GES-1) and observed that both CDCA and DCA significantly elevated the expression of intestinal biomarkers, including CDX2, Kruppel-like factor 4 (KLF4), MUC2, and proteins associated with chorionic villi, at both gene and protein levels. This observation conclusively demonstrates that BAs stimulation can induce an IM phenotype in gastric epithelial cells (Xu C. et al., 2020). Complementing these findings, Yu et al. reported that exposure to DCA, CDCA, or their combinations markedly increased the levels of CDX2 and MUC2 in the gastric mucosa of mice, thereby substantiating the crucial role of BAs in the induction of gastric intestinal metaplasia (GIM) (Yu et al., 2019). Another investigation highlighted the pivotal role of bile reflux in the development of GIM, noting that DCA-induced GIM enhances the digestion and absorption of gastric fats while concurrently diminishing gastric acid secretion. Furthermore, In the stomach, DCA reduced microbial diversity but promoted the abundance of several bacterial genera, such as the Rikenellaceae RC9 gut group. It was shown that the Rikenellaceae RC9 intestinal group was significantly positively correlated with DCA and RGD1311575 (capping protein regulator of inhibition of actin dynamics) and that RGD1311575 was positively correlated with Fabp1 (fatty acid binding protein, liver). Ultimately, the study identified the DCA-Rikenellaceae RC9 intestinal group-RGD1311575/Fabp1 axis as a potential key mechanism in the pathology of bile reflux-associated GIM (Xu et al., 2023).

Recent studies have validated the therapeutic potential of Xiaojianzhong Tang (XJZ) for treating CAG. This traditional formulation showed significant modulation of the BAs metabolites CA, DCA, glycodeoxycholic acid, taurodeoxycholic acid, docosahexaenoic acid, and L-isoleucine. Notably, comprehensive analyses through 16S rRNA gene sequencing have indicated that XJZ significantly mitigates disruptions in the intestinal microbiota of rats afflicted with CAG, with a marked influence on bacteria associated with BAs metabolism. This includes an array of bacteria such as Butyric acid bacteria, Desulfovibrio vulnificus, *Lactobacillus*, *Lactobacillus* parahippuricus, *Acetobacter*, and *Lactobacillus* acidophilus. Moreover, molecular docking assessments underscore the proficient binding affinity of XJZ-regulated metabolites to BAs-related molecular targets, thereby substantiating the role of XJZ in modulating both BAs-related microbial populations and metabolic pathways, which are critical for the management of CAG (Liu et al., 2023a). Simultaneously, the role of cinnamon in CAG treatment has been extensively studied, owing to its anti-inflammatory, antioxidant, and anti-tumor properties. Investigations have illustrated that BAs metabolites significantly contribute to the therapeutic efficacy of cinnamon in CAG management. Experiments involving sodium deoxycholate intervention in rat models of CAG have shown a pronounced inhibition of primary BAs synthesis. This intervention leads to a decrease in BAs concentrations and an increase in DCA and its derivatives, further confirming that the modulation of primary BAs biosynthesis is a pivotal mechanism by which cinnamon exerts its therapeutic effects in CAG (Liu et al., 2023b).

Previous studies have described resveratrol as a compound that has multiple functions such as antioxidant, anti-inflammatory and anti-tumor effects. The current studies have shown that resveratrol increases phosphorylation and translocation of FoxO4 into the cytoplasm and thus activating it, while at the same time decreasing CDX2 expression through the PI3K/AKT signaling pathway. It is also revealed that the specific knockdown of endogenous FoxO4 not only enhances its phosphorylation level but also attenuates the upregulation of GIM markers by CDCA. These outcomes together provide the evidence that resveratrol could ameliorate BAs- induced GIM through the modulation of PI3K/AKT/p-FoxO4 signaling pathway, thereby suggesting its therapeutic potential for the reversal of pathological process of GIM (Lu et al., 2021). In parallel, Modified Chaishao Liujunzi Decoction (MCLD), a renowned Chinese herbal formulation, has been utilized to effectively manage clinical symptoms, alleviate gastric mucosal inflammation, and promote healing of gastric mucosal lesions among patients with GIM. It has been demonstrated that MCLD robustly curtails DCA-stimulated cellular proliferation and diminishes the expression of pro-inflammatory cytokines as well as gut-specific markers. Moreover, MCLD exerts a negative regulatory effect on the expression of genes and proteins associated with the EGFR/PI3K/AKT/mTOR pathway, thereby substantiating its therapeutic potential to ameliorate BAs-induced GIM *via* this signaling pathway (Sun et al., 2023).

## 3 Progress of research on gut microbiota in the context of PLGC

### 3.1 Overview of gut microbiota regulatory mechanisms

The human gastrointestinal tract harbors a complex and diverse community of microorganisms that are essential for maintaining physiological homeostasis and regulating numerous bodily functions in both states of health and disease (de Vos et al., 2022; Jandl et al., 2024; Kim et al., 2024; Noecker and Turnbaugh, 2024). These intestinal microbiota metabolize dietary components, resulting in the production of a wide array of active microbial metabolites, which vary based on dietary composition. The generation of these beneficial metabolites is contingent upon the presence and resilience of advantageous microorganisms, illustrating the dynamic interplay between the host’s immune system and the gut microbiota. Moreover, the co-evolution of humans and their microbiota seems to facilitate the creation of microbial metabolites that are structurally suited to interact with specific host receptors, thereby influencing the host’s physiological responses (Cohen et al., 2017). Macrogenomics, macrotranscriptomics, and metabolomics have been developed in the last few years and this has provided information on many microbial metabolites with the genes that code for their production (Li and Han, 2024). According to the current research, the microorganisms present in the human gut are known to synthesize about 50,000 different microbial metabolites. Of these, approximately 22,500 are thought to have anti-bacterial activity. Besides their antimicrobial actions, these metabolites modulate the intestinal epithelial cells (IECs) and immune cells in the gut. This interaction is very vital in modulating the health of the host by enhancing the efficiency of the gut barrier and hence plays a crucial role in the overall health of the gastrointestinal tract (Kho and Lal, 2018; Li et al., 2018).

**FIGURE 2:**
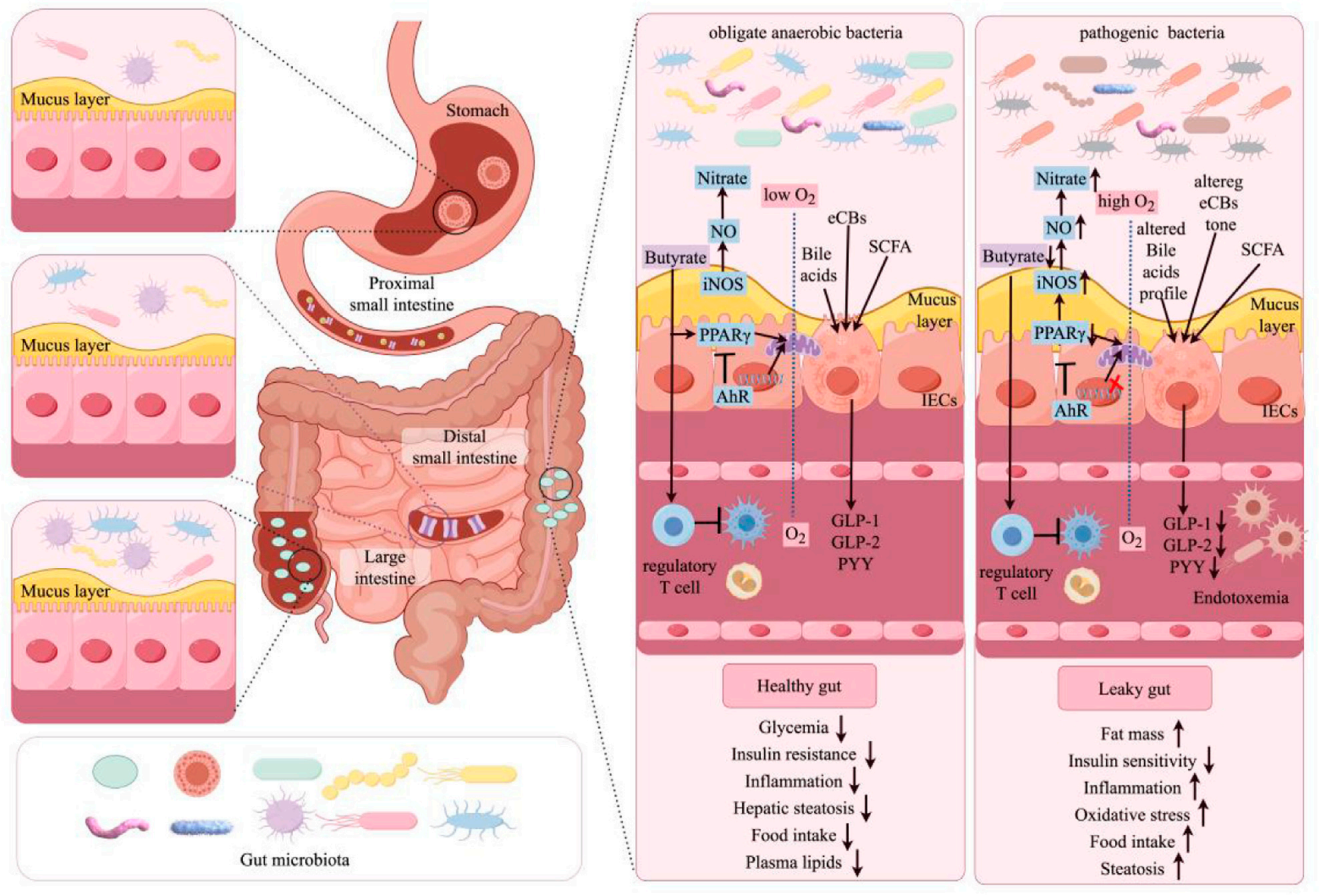
Molecular regulatory mechanisms of the host gut microbiota in healthy and pathological conditions. NO, nitric oxide; iNOS, inducible nitric oxide synthase; eCBs, endocannabinoids; SCFA, short-chain fatty acids; IECs, intestinal epithelial cells; AhR, aryl hydrocarbon receptor; GLP-1, glucagon-like peptide-1; GLP-2, glucagon-like peptide-2; PYY, Peptide YY.

Some of the most important molecular pathways that link the gut microbiota with the host health are as follows (Figure 2). At the best of health, colonocytes metabolise butyrate as their preferred energy source through the mitochondria’s β oxidation pathways. This metabolic pathway not only uses oxygen but also fosters conditions of anaerobic respiration within the gut lumen where it is fundamental for the good health of the gastrointestinal tract. In addition, butyrate is well known to bind the peroxisome proliferator-activated receptor gamma (PPARγ) and in doing so, modulate the activity of the inducible nitric oxide synthase (iNOS). This suppression reduces the production of nitric oxide and therefore reduces the production of nitrate which is important in controlling the levels of oxidative stress in the colon. On the other hand, the reduction of luminal butyrate concentrations in pathological states is associated with lower activity of PPARγ. This decrease in PPARγ activity is connected with activation of glycolytic processes and reduction of oxygen consumption rate, which improves conditions for iNOS overexpression. This increase in iNOS will lead to higher production of NO which in turn will lead to higher availability of nitrates that will be advantageous to pathogenic bacteria that may be growing in such conditions. Also, butyrate has a significant function in immune regulation as it promotes the regulatory T cells to lessen the inflammation. This is important to help in the regulation of the immune system and avoid the over activation of inflammatory markers which can cause tissue injury. AhR is a nuclear transcription factor that binds to DNA and regulates gene expression. Under normal circumstances, the nuclear transcription factor AhR is overexpressed and active in colonocytes to modulate cellular response to xenobiotics or dietary factors. Nevertheless, reduced activity of AhR, which could be attributed to inadequate interaction with AhR agonists, results in alterations in the intestinal barrier function and this is critical to maintaining gut health. In addition, enteroendocrine cells contain essential receptors that recognize short-chain fatty acids, some endogenous cannabinoids, and BAs. Activation of these receptors significantly enhances the secretion of important intestinal peptides, such as GLP-2, GLP-1, and peptide YY. These peptides play a significant role in reducing intestinal permeability, improving insulin secretion and sensitivity, lowering food intake, and decreasing plasma lipid levels. Furthermore, these elements play a crucial role in mitigating hepatic steatosis and metabolic endotoxemia, both conditions linked to decreased inflammatory responses. This interaction between gut microbiota and these molecular mediators thus helps to fortify the gastrointestinal barrier, enhance metabolic health, and mitigate inflammatory states. In contrast, pathological conditions are marked by the reverse effects, leading to compromised gut health and increased disease susceptibility (Rastelli et al., 2019).

### 3.2 Gut microbiota regulation of PLGC mechanisms

CDX2 is actively expressed within the chemotactic mucosa of the human intestine and has been observed to induce IM in the stomachs of CDX2 transgenic mice, illustrating its significant role in gastrointestinal cellular transformation (Barros et al., 2012). Detailed microbial analysis has shown that groups such as the *Clostridium difficile* group, the *C. difficile* subgroup, the Anaplasma fragilis group, and Prevotella spp. were absent in the jejunum or ileum of typical mice. However, these groups were identified in CDX2 transgenic mice, underscoring notable differences in the microbial populations between standard and genetically modified mice. This might be due to reduced production of gastric acid and the development of IM in the CDX2 transgenic mice and thus a clear shift in the gut microbiota composition (Sakamoto et al., 2015). The further studies of the composition of the microbiome in the stomach with IM have shown that J. ignava and Filifactor alocis are overrepresented in the stomachs of affected animals. On the other hand, The fungal strains Coccidioides variegatus, C. parasiticus and C. discoideum were reported to be less frequent in subjects with IM as compared to the healthy controls. This pattern of microbial distribution supports the hypothesis that some elements of oral and gastric microbiota are involved in the pathogenesis of PLGC. These microbial entities are involved in the regulation of the inflammatory pathways and therefore, it can be deduced that certain bacterial profiles are associated with the regulation of inflammation in gastric pathologies (Wu et al., 2022).

From the most recent experimental works, it has been revealed that *H. pylori* infection reduced, to a great extent, the complexity and richness of gastric microbiota especially in the phases of IM and GC, though the impact is not so significant in the CAG phase (Shirani et al., 2024; Xiao and Ma, 2022). Also, the number of species as well as the overall density of the gastric microbiota were also found to be much lower in *H. pylori* infected patients. The most interesting finding in this regard is that the densities of several bacterial species including *Neisseria*, *Streptococcus*, Verticillium, and Veillonella are found to be significantly higher in *H. pylori*-positive IM cases as compared to chronic superficial gastritis; *Neisseria* showed the maximum increase (Liu et al., 2022a). In particular, more detailed research of the mucosal microbiota of patients diagnosed with IM and GC has shown that there are marked changes. These disturbances are associated with enhanced microbial activity in the gastric niche of *H. pylori* negative patients as compared to the *H. pylori* positive ones. This increased anastomosis of the gastric microorganisms in the absence of *H. pylori* points towards a form of microbial community response. This research also provides evidence for the idea that some microorganisms such as *P. stomatis*, *D. pneumosintes*, *S. exigua*, *P. micra*, and *S. anginosus* may be involved in the development and worsening of IM and GC and may affect the disease natural course and treatment response (Coker et al., 2018).

The herb Huangqi (HQ), is one of the most commonly used Traditional Chinese Medicine in the management of CAG (Zhang R. et al., 2024). Previous investigations have established that HQ greatly alters the structure of the gut microbiota and metabolic characteristics in the rat model of CAG. Especially, the treatment with HQ was established to influence several vital metabolites and 7-keto-3A-12-α-hydroxyalkanoic acid and DCA were the most affected metabolites. These alterations are suggestive of HQ’s capacity to not only alter the microbial profile of the intestinal tract but also to overcome the adverse impact that CAG imposes on the microbial metabolism and thus provide protection to the intestinal microbiota (Liu et al., 2022b). This broad modulation of both microbial and metabolic variables indicates that HQ’s therapeutic effects go beyond the traditional treatment modalities to also target the bio-ecological changes that are characteristic of CAG. HQ helps in maintaining the balance of the gut microbiota and the metabolic disturbance associated with this gastric condition thus offering a complete solution for managing the related complications.

Chai Shao Liu Jun Zi decoction (CSLJZD) is a well-recognized herbal formula that provides cure to CAG. A previous study conducted between CAG patients and healthy participants revealed that the microbial richness and diversity, as measured by Chao1 and ACE indices are significantly higher in CAG patients. Interestingly, these indices were found to have been reduced considerably in the patients who were given oral administration of the herbal formula known as CSLJZD together with a vitamin cofactor in treating CAG (Xing et al., 2024). In addition, the treatment group had a high significant change in some microbial genera such as *Klebsiella* spp. Some of the predominant taxa include Proteobacteria phylum and specifically Deltaproteobacteria, and Gemmiger spp. This increase in microbial counts can be considered as a strong indicator of CSLJZD’s efficacy in the management of CAG affected patients’ gut microbiota. Qinghuayin (QHY) is another important traditional herbal formula which has been used in the treatment of CAG. Further analysis of its effects shows that use of QHY leads to an increase in the total bacterial load including lactobacilli in the GI tract. Imbalance of microbial flora is an unfavorable side effect of CAG and the use of QHY is known to be effective in not only combating this problem but also in promoting a more effective equilibrium by greatly improving the microbial diversity in the intestines. This increase in the microbial diversity is useful in enhancing the health of the gut which in turn reduces the impacts of CAG (Huang et al., 2021).

## 4 Mechanism of crosstalk between BAs and gut microbiota and its role in PLGC

**FIGURE 3:**
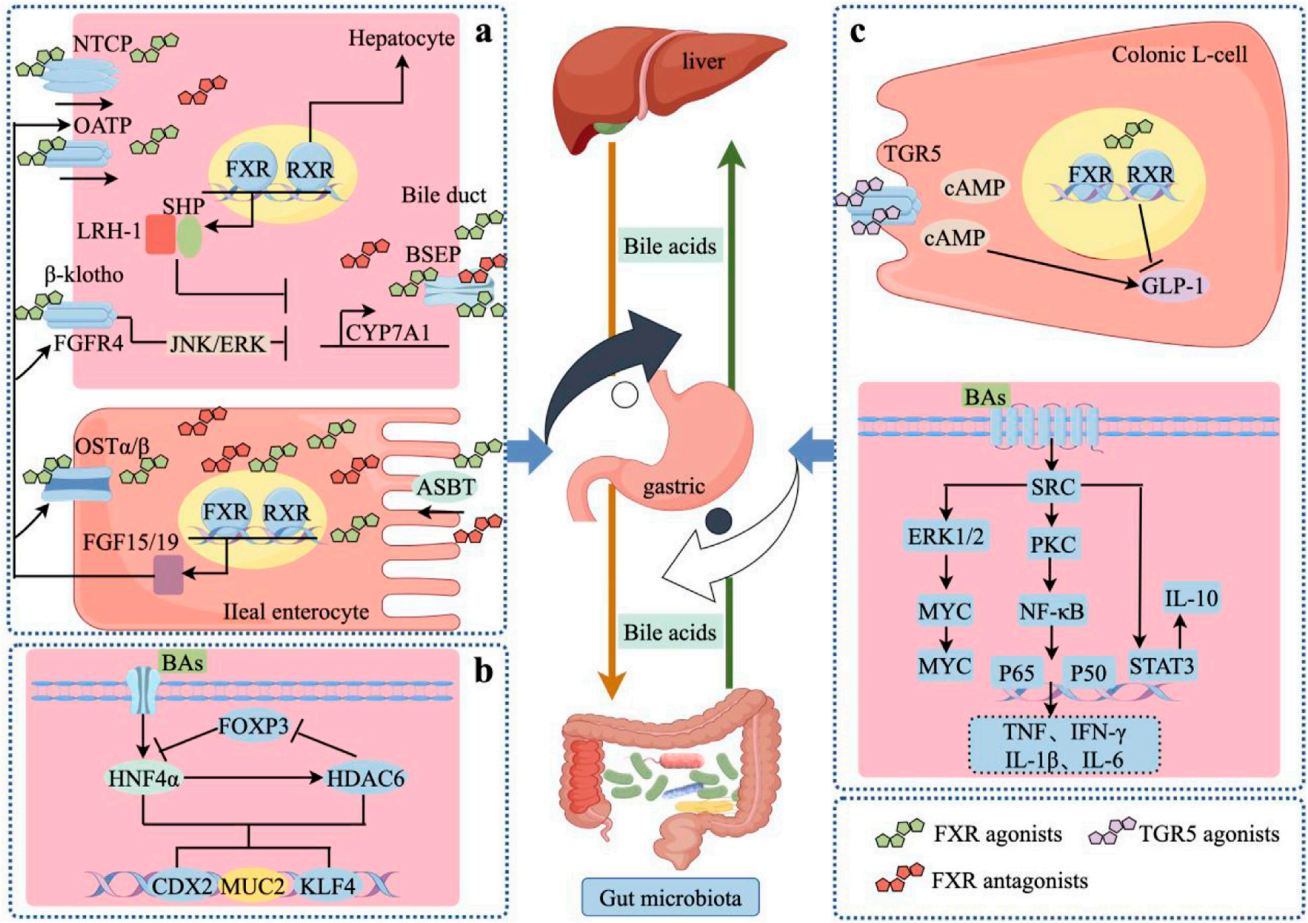
BAs and gut microbial crosstalk mechanisms. **(a)** FXR receptor-mediated crosstalk mechanisms regulate PLGC; **(b)** HDAC6/HNF4α-mediated crosstalk mechanisms regulate PLGC; **(c)** TGR5 receptor-mediated crosstalk mechanism regulates PLGC. NTCP, sodium taurocholate cotransporting polypeptide; OATP, organic anion transporting polypeptide; FXR, farnesoid X receptor; RXR, retinoid X receptor; SHP, recombinant small heterodimer partner; LRH-1, liver receptor homolog 1; BSEP, bile salt export pump; FGFR4, fibroblast growth factor receptor 4; JNK, c-Jun N-terminal kinase; ERK, extracellular regulated protein kinases; OSTα/β, Organic Solute Transporter alpha/beta; ASBT, apical sodium-dependent bile acid.

The intricate interplay between BAs and the gut microbiota critically influences the composition of the microbiota and provides defense mechanisms against intestinal pathogens (Lee et al., 2024; Ridlon and Gaskins, 2024). Through the modulation of BAs, the microbiota supports the maintenance and regeneration of the gut barrier while orchestrating the development of both innate and adaptive immune responses in the host. Moreover, metabolites derived from BAs offer protection against several opportunistic pathogens, and bacteria that have colonized the intestines can disrupt the endocrine regulation of BAs absorption and secretion in the ileum (Chang, 2020; Ghosh et al., 2021; Uribe et al., 2016). BAs engage with a diverse array of receptors to modulate various physiological pathways. Distinct BAs demonstrate varying affinities for these receptors; for instance, Primary bile acids CA and CDCA are potent ligands for FXR, whereas DCA and lithocholic acid are potent ligands for TGR5 (Qi et al., 2022; Taylor et al., 2024; Wang S. T. et al., 2024; Xiang et al., 2023) (Figure 3).

### 4.1 FXR receptor-mediated crosstalk regulation of PLGCs

BAs act as agonists for the FXR, exhibiting varying levels of activity based on their structural differences. Among them, CDCA stands as the most effective FXR agonist, followed by DCA, LCA, and CA. While the specific impacts of these interactions on FXR functionality remain less understood, it has been observed that bound BAs can activate FXR in the small intestine at concentrations up to 10 mM (Inagaki et al., 2006; Shin and Wang, 2019). Additionally, FXR plays a crucial role in overseeing various steps in the synthesis of primary BAs such as CA and CDCA. Conversely, a deficiency in FXR activity in mice results in metabolic disturbances and a compromised regulation of host BAs levels (Ding et al., 2024; Gioiello et al., 2024). Research also indicates that the expression of FXR within the intestinal system is essential for maintaining normal cellular functions and preventing tumorigenesis. In contrast, abnormal expression of FXR in the stomach is linked to an increased risk of developing PLGC and GC (Li et al., 2015).

In hepatocytes, BAs activate FXR, leading to SHP induction, which interacts with liver receptor homolog-1 to inhibit CYP7A1 gene expression, essential for BAs synthesis (Yu Cai Lim and Kiat Ho, 2024). In enterocytes, FXR activation by primary BAs regulates BAs synthesis, transportation, secretion, and absorption (Jiang et al., 2021). FXR, when dimerized with RXR, promotes transcription of genes for BAs transcellular movement and FGF19 (FGF15 in mice) regulation (Díaz-Holguín et al., 2023; Kliewer and Mangelsdorf, 2015). These proteins, once in the portal circulation, activate the liver’s FGF receptor 4/β-Klotho complex, leading to phosphorylation of ERK 1/2 and subsequent suppression of CYP7A1 gene expression through interactions with HNF4α and liver receptor homolog-1, thus reducing BAs synthesis (Fiorucci et al., 2021).

CDX2 is a transcription factor that is typically specific to the gastrointestinal tract, with its expression normally confined to this region. However, CDX2 is found to be expressed at elevated levels in tissues affected by GIM (Dawson and Karpen, 2015; Xie et al., 2024). Research conducted by Yu et al. (2019) demonstrates that components of BAs, specifically DCA and CDCA, enhance the expression of CDX2 and MUC2 *via* the FXR/NF-κB signaling pathway. This activation plays a crucial role in disrupting the equilibrium of the gut microbiota, thereby fostering the progression of GIM. Additionally, findings by Li et al. (2019) indicate that the reflux of CDCA activates FXR, leading to an increased expression of miR-92a-1-5p. This microRNA downregulates FOXD1, negating its suppressive impact on the NF-κB pathway. The activation of this pathway subsequently promotes the transcription of CDX2 and intestinal differentiation, further destabilizing the balance of the intestinal microbiota and contributing to the development of PLGC. Members of the miR-17-92 cluster are recognized for their elevated expression in various forms of cancer and have been identified at significantly higher levels in the sera of patients with GC and IM. This observation has led to the exploration of miR-92a as a viable serum biomarker for the early detection of gastric cancer, due to its potential predictive value in clinical diagnostics (Fan et al., 2018; Li et al., 2017; Liu et al., 2018; Sun et al., 2020). In detailed studies, Yuan et al. (Yuan et al., 2019) have discovered that DCA diminishes SOX2 expression by inducing miR-21, which binds directly to the 3′-UTR of the gene. Concurrently, DCA promotes the expression of CDX2 in gastric cancer cells, illustrating a complex interplay between these molecules that affects cellular behavior in cancerous tissues. Further compounding the impact of SOX2, its overexpression is known to significantly repress the transcription of genes that are critical for intestinal epithelial chemotaxis and the formation of the SOX2-CDX2 protein complex, which plays a crucial role in inhibiting CDX2 function. However, the strategic knockdown of miR-21 can alleviate the suppressive effects of SOX2 on these pathways, thereby enhancing the stability and health of the intestinal microbiota, and offering innovative approaches to the prevention of PLGC. Additionally, pioneering research by Pyo et al. (Pyo et al., 2015) has revealed that physiological concentrations of DCA can stimulate the expression of MUC2 in gastric cancer cells, while simultaneously reducing the expression of Snail, a key regulator of epithelial-mesenchymal transition. This activity is accompanied by an increase in E-calmodulin expression and alterations to the species and composition of the intestinal microbiota. These molecular modifications are crucial for inhibiting tumor invasion and migration, highlighting the critical importance of maintaining BAs concentrations within physiological thresholds to prevent the progression of gastric cancer and the development of precancerous lesions known as PLGC.

Substantial evidence supports that small heterodimer partner (SHP) is a key downstream gene regulated by FXR in BAs biosynthesis. Zhou et al. (2018) observed in both *in vivo* and *in vitro* studies with normal PKGC and GC cell lines that BAs enhances the transcriptional expression of SHP *via* FXR activation. This enhancement subsequently elevates CDX2 expression, which influences the abundance of gut microbiota and plays a significant role in the progression of GIM. Moreover, the research noted that FXR expression levels were distinctly higher in GC cell lines compared to normal cells. XJZ has been proven effective in treating CAG, with research confirming that XJZ regulates disturbances in the intestinal microbiota of CAG rats. Notably, it influences bacteria associated with BAs metabolism, including Butyric acid bacteria, Desulfovibrio vulnificus, *Lactobacillus*, *Lactobacillus* parahaemolyticus, *Acetobacter*, and Acidophilus (Choudhuri and Klaassen, 2022). This modulation suggests XJZ’s potential in restoring microbial balance, highlighting its therapeutic efficacy in managing conditions linked to disrupted BAs pathways.

### 4.2 TGR5 receptor-mediated crosstalk regulation of PLGCs

TGR5, recognized as a receptor specific to BAs and belonging to the G protein-coupled receptor family, is universally expressed across human and animal tissues. This receptor initiates a range of intracellular signaling cascades when it interacts with BAs (Ay et al., 2024; Jia et al., 2018; Papadopoulos et al., 2024). This receptor is predominantly stimulated by secondary BAs such as LCA and DCA, making it a focal point in studies exploring the interactions between the microbiota and BAs.

Activation of TGR5 triggers the stimulation of adenylate cyclase, leading to the production of cAMP and subsequent activation of protein kinase A. This cascade of biochemical events regulates various inflammatory and metabolic processes, including BAs homeostasis, glucagon-like peptide-1 (GLP-1) production, insulin sensitivity, and energy metabolism (Ye et al., 2024). In comparisons, TGR5 demonstrates a higher affinity for secondary BAs than for bourbonic acid, with taurine-conjugated BAs showing greater potency at the TGR5 receptor than either unconjugated or glycine-conjugated BAs. Research findings have revealed that mice undergoing vertical sleeve gastrectomy (VSG) exhibit an increase in total serum BAs levels and a reduction in intestinal lipid absorption. This surgical intervention also leads to the downregulation of CYP8B1, which results in a lower ratio of 12α-hydroxylated to non-12α-hydroxylated BAs. Post-operatively, modifications in the gut microbiota following VSG contribute to a decrease in the inflammatory responses within the gastric mucosa, thereby decelerating the progression of PLGC (Tu et al., 2022).

Carino et al. (2016) conducted immunohistochemical and R-PCR analyses which demonstrated that TGR5, a membrane-bound BAs receptor, is activated by taurocholic acid and is significantly expressed in the tissues of patients with PLGC and in the late stages of gastric cancer (stages III and IV). The expression of TGR5 was found to be associated with an increase in N-calmodulin levels and the acquisition of an invasive phenotype. Building on this, Ni et al. (2020) provided the first evidence that, under the influence of DCA, TGR5 activates the TGR5-ERK1/2 signaling pathway. This activation leads to the induction of HNF4α expression, which in turn directly upregulates the expression of KLF4 and CDX2. These changes have significant implications on the balance of the intestinal microbiota, thereby fostering the progression of PLGC. Therefore, targeting the TGR5-HNF4α signaling cascade for inhibition may present a viable therapeutic approach to curtail the development of PLGC and gastric cancer.

An experimental study proved that in both tissue culture model and animal model DCA activates STAT3 signaling pathways and regulates the expression of the IM marker KLF5 in mouse gastric organ tissues. In models of INS-GAS mice, DCA was revealed to enhance the level of total serum BAs and to accelerate the development of gastric I/M and dysplasia. Furthermore, DCA caused significant alterations in the gastric environment which included abnormal BAs metabolism and disturbance in the balance of the gut microbiota. This also included a particular increase in the abundance of the genera Gemmobacter and *Lactobacillus*. These changes substantiate the proposal that DCA’s activation of nuclear STAT3 phosphorylation is a primary regulation mechanism for increased KLF5. This mechanism is linked with the initiation of gastric inflammation and the progress of IM which implies that DCA affects both the gut microbiota and gastric microbiota as well as the general balance between the gut microbiota and gastric microbiota, and BAs metabolism (Jin et al., 2022).

### 4.3 HDAC6/HNF4α-mediated crosstalk regulation of PLGCs

Histone deacetylase 6 (HDAC6) has been referred for enhancing cell proliferation and was observed to be over expressed in the IM and GC tissues of *H. pylori* positive patients and can therefore be involved in the regulation of intestinal epithelial chemotaxis (Song et al., 2023; Xu X. et al., 2020). Also, it has been reported that hepatocyte nuclear factor 4 alpha (HNF4α), a transcription factor involved in the regulation of liver and intestinal cell differentiation and development (Qiu et al., 2024), is associated with HDAC6 to form a regulatory feedback mechanism. This interaction enables the sequential development of IM governed by the activity of miR-1, thus pointing to a network of molecular interactions involved in the development of gastric diseases.

Studies have shown that BAs inhibit the histone deacetylase 6 (HDAC6) that in turn affects the forkhead box protein P3 (FOXP3) whose product represses the hepatocyte nuclear factor 4 alpha (HNF4α). This suppression is useful in the encouragement of gastric IM. It has been seen that the level of miR-1 is reduced when treated with BAs and this led to increased levels of HNF4α and HDAC6. These alterations drastically affect the profile of the intestinal microbiota and its density; this in turn increases the expression of the HDAC6 promoter. Also, this work provides evidence that HDAC6 controls the expression of FOXP3 through epigenetic regulation mechanisms. Consequently, FOXP3 inhibits the transcription of HNF4α establishing a regulatory feed-forward loop that enables activation of intestinal markers and formation of IM. This feedback loop of HDAC6, FOXP3, and HNF4α show that the pathogenesis of intestinal metaplasia is controlled by a intricate regulatory network (Zhang et al., 2022).

A previous study has revealed that BAs are vital in the growth of gastric IM through the regulation of transcription factors including KLF4 and CDX2. After BAs treatment, HNF4α is upregulated which in turn activate the TGR5-ERK1/2 signaling pathway. This activation greatly upregulates chemotactic markers and directly modulates the expression of KLF4 and CDX2 which leads to the imbalance of the gut microbiota (Ni et al., 2020). In addition, the study identified that both gastric IM cell models and patient specimens had high expression of HDAC6 and HNF4α, and HNF4α can bind to the promoter of HDAC6 to activate its transcription, which in turn affects cell behaviour. On the other hand, a significant increase of HDAC6 activity was observed to enhance HNF4α protein level in GES-1 cells. This interaction also points towards the fact that HDAC6 besides playing a role in histone acetylation also has an effect on protein turnover and function. Furthermore, microRNA-1 have been demonstrated to have an imperative role in this regulatory network through targeting of HDAC6 and HNF4α thus suppressing downstream markers of intestinal differentiation. This suppression is important to control the development and the severity of gastric intestinal metaplasia, thus underlining the multifactorial genetic and epigenetic based of this disease (Wang et al., 2021).

## 5 Conclusion

This study posits that BAs act as a critical pathogenic factor in the development of PLGC by inducing injuries to the gastric mucosa. Concurrently, disturbances in the gut microbiota represent a significant pathological manifestation associated with these mucosal injuries. Consequently, the prevention of bile reflux and the strategic modulation of the gut microbiota are underscored as crucial interventions to mitigate mucosal damage in PLGC and to impede tumor progression. This approach is particularly vital through the mechanism of crosstalk that regulates the interaction between BAs and the gut microbiota. It has been observed that the gut microbiota plays an integral role in the biotransformation and modification of BAs. Inversely, the composition of BAs has been found to influence the proliferation of the gut microbiota. Furthermore, addressing metabolic imbalances between BAs and the gut microbiota, particularly through the interactions mediated by the FXR receptor, the TGR5 receptor, and the HDAC6/HNF4α axis, can effectively delay the progression and alleviate the condition of PLGC.

Our study through inductive analysis has determined that under normal physiological conditions, BAs influence the composition of the microbiota *via* their antimicrobial properties and by activating host signaling pathways essential for maintaining intestinal homeostasis. Moreover, the intestinal microbiota enhances the BAs pool through oxidation and specific isomerization at the C-3, C-6, C-7, and C-12 positions on sterols, facilitating mutual protection against gastric mucosal damage. Under pathological scenarios, BAs reflux into the stomach compromises the structural integrity of the gastric mucosa and disrupts gastric microorganisms. This leads to a dysregulation of the intestinal microbiota, abnormal BAs metabolism, and a disrupted metabolic balance between BAs and the intestinal microbiota, thereby accelerating the onset and progression of PLGC. Furthermore, natural active pharmaceutical ingredients and compounds play a crucial role in managing the balance between BAs and intestinal microbiota by modulating their crosstalk pathways. This regulation not only alleviates gastric mucosal inflammation but also ameliorates gastric mucosal lesions, thereby contributing to the prevention and treatment of PLGC.

The pathogenesis of PLGC is recognized for its complexity, focusing on various aspects such as the inflammatory microenvironment, autophagy, BAs, apoptosis, and glycolysis. Recent insights also point to significant roles for gut microbiota and epigenetic modifications like DNA methylation in the progression of PLGC. Despite the broad spectrum of factors involved, current research often remains isolated to individual pathogenic mechanisms, with a notable absence of studies that integrate multi-mechanistic crosstalk and the association of multiple targets. Such investigations are still largely in their nascent stages. This paper proposes a novel pathway and target for exploring the crosstalk between BAs and gut microbiota, aiming to offer fresh theoretical insights and innovative approaches for PLGC treatment. The objective is to extend beyond the traditional single-pathway focus and encourage a more holistic view of the interconnections between various biological systems influencing PLGC.

In forthcoming studies, it is pivotal that the focus be placed on the BAs -gut microbiota interaction pathway, identifying and targeting its critical components to modulate the intestinal flora balance and curb bile reflux. By either promoting or inhibiting specific processes within this pathway, the functionality of the gastric mucosa can be safeguarded, thus contributing significantly to the prevention and treatment of PLGC. Concurrently, building upon current pharmacological treatments, further investigations should delve into the molecular mechanisms by which drug interventions affect the BAs-gut microbiota pathway, particularly through the multifaceted impacts of active compounds found in traditional Chinese medicines. Moreover, the research should persist in uncovering potential targets and signaling pathways involved in the multifaceted crosstalk regulation of PLGC. The aim would be to design highly effective, low-toxicity therapeutic drugs that are finely tuned to these targets, thereby furnishing new clinical evidence for drug development and application. In sum, the vision for future research is to delineate precise, effective, and well-founded strategies and tools for combating PLGC.
